# A network-based drug repurposing method via non-negative matrix factorization

**DOI:** 10.1093/bioinformatics/btab826

**Published:** 2021-12-07

**Authors:** Shaghayegh Sadeghi, Jianguo Lu, Alioune Ngom

**Affiliations:** School of Computer Science, University of Windsor, 401 Sunset Avenue, N9B 3P4, Windsor, Ontario, Canada; School of Computer Science, University of Windsor, 401 Sunset Avenue, N9B 3P4, Windsor, Ontario, Canada; School of Computer Science, University of Windsor, 401 Sunset Avenue, N9B 3P4, Windsor, Ontario, Canada

## Abstract

**Motivation:**

Drug repurposing is a potential alternative to the traditional drug discovery process. Drug repurposing can be formulated as a recommender system that recommends novel indications for available drugs based on known drug-disease associations. This article presents a method based on non-negative matrix factorization (NMF-DR) to predict the drug-related candidate disease indications. This work proposes a recommender system-based method for drug repurposing to predict novel drug indications by integrating drug and diseases related data sources. For this purpose, this framework first integrates two types of disease similarities, the associations between drugs and diseases, and the various similarities between drugs from different views to make a heterogeneous drug–disease interaction network. Then, an improved non-negative matrix factorization-based method is proposed to complete the drug–disease adjacency matrix with predicted scores for unknown drug–disease pairs.

**Results:**

The comprehensive experimental results show that NMF-DR achieves superior prediction performance when compared with several existing methods for drug–disease association prediction.

**Availability and implementation:**

The program is available at https://github.com/sshaghayeghs/NMF-DR.

**Supplementary information:**

Supplementary data are available at *Bioinformatics* online.

## 1 Introduction

Drug repurposing (DR) is based on the idea that drugs can be used for treating disease conditions other than the initial target of treatment (Serçinoğlu and Sarica, 2019). DR has become a widespread tactic as this strategy is efficient, cost-effective and a low-risk alternative for drug discovery (Zhou *et al.*, 2020). Computational drug repurposing (CDR) aims to find novel indications for existing drugs to treat diseases other than the drugs’ original purpose via using computational methods such as machine learning (Shim and Liu, 2014). Compared with the experimental means, CDR methods are more efficient in providing treatment solutions for all diseases such as rare, acute, neglected diseases and cancer indications (Hernandez *et al.*, 2017). Besides, in epidemic disease outbreaks such as COVID-19, the use of derivatives of previously known antiviral drugs is a useful strategy (Dotolo *et al.*, 2021; Shah *et al.*, 2020; Wang, 2020).

To date, a variety of computational methods have been proposed to solve these problems and to predict new interactions between known drugs and diseases accurately. They fall into two categories (i) network-based and (ii) learning-based (Supplementary Fig. S1). However, these methods still suffer from various computational challenges such as handling heterogeneous data, scalability and class imbalance for learning-based methods and sparsity for network-based methods. To overcome these limitations, we propose in this work a novel network-based method (Non-Negative Matrix Factorization-based Drug Repurposing) NMF-DR, to accurately predict potential new drug–disease interactions.

CDR can also be modeled as a recommendation system that recommends the top-ranked diseases for given drugs (Luo *et al.*, 2018a). Recommendation systems nicely model this problem and also are able to deal with existing challenges in CDR. There are network-based DR methods that have been presented to infer new drug–disease interactions with modeling associations between biological concepts in biological networks. Network-based DR can be classified into two approaches, i.e. random walk (RW) approach; such as RWHNDR (Luo *et al.*, 2018a), TL-HGBI (Wang *et al.*, 2014b), MBiRW (Luo *et al.*, 2016); and matrix factorization (MF) approach; such as DRRS (Luo *et al.*, 2018a), KBMF (Gönen *et al.*, 2013), MSBMF (Yang *et al.*, 2021), SCPMF(Meng *et al.*, 2021). RW approach is more scalable and popular, but MF approach achieves higher accuracy (Luo *et al.*, 2018a).There are also other MF-based methods which instead of drug-disease networks, they use drug-protein network for DR purpose such as Ceddia *et al.* (2020), Ceddia *et al.* (2019) and Dissez *et al.* (2019).

The network-based strategy is more successful in increasingly attracting attention from the pharmaceutical community in recent years. Also, due to the advances of high-throughput technology and an increasing number of available data sources (e.g. genetic, pharmacogenomics, clinical, chemical agent, etc.), this approach can be broadly used in the CDR field (Luo *et al.*, 2018a). As for Learning-based methods, there are some successful methods. Most of the well-known learning-based methods, such as PREDICT(Gottlieb *et al.*, 2011), PreDR(Wang *et al.*, 2013), SMKF(Moghadam *et al.*, 2016), focus on the prepossessing steps and try different kernel-based integrating methods for fusing drug–drug similarities and disease–disease similarities.

Network-based methods and MF-based methods have the following advantages that make them stand out. In the DR problem, we have more unknown interactions than known interactions between drugs and disease. This issue can lead to the class-imbalance problem in learning-based approaches and sparsity in network-based approaches. In a network-based approach, unknown interactions are treated as unlabeled associations instead of negative associations. This helps avoid the problem of wrong label assignment, which would affect the performance of models to some extent (Shahreza *et al.*, 2017).

MF-based methods provide a principled framework for the integration of heterogeneous network data (Gligorijević and Pržulj, 2015). In addition, these methods show higher accuracy than learning-based methods (Sadeghi and Keyvanpour, 2019b). As a result, the CDR method proposed in this article, a network-based MF method, is proposed to deal with sparsity, heterogeneous data and scalability, which are some of the most critical challenges in drug repurposing problem (Sadeghi and Keyvanpour, 2020).

Hence, in this study, we propose an improved network-based MF-based model called NMF-DR. The novelty of this model can be explained based on its two phase:

1. Pre-Processing phase: (I) we propose a method for analyzing and normalization of similarity matrices; (II) we propose a weighting approach for constructing a heterogeneous network of drugs and diseases.
2. Relation Prediction Phase: for estimating the missing values of links between drugs and diseases, we propose an improved non-negative matrix factorization (NMF) method that contains (I) a rank selection method based on minimum descriptive length (MDL) methods; (II) a matrix initialization method based on singular value decomposition (SVD) methods; (III) an accelerated NMF method for relation prediction step.

The rest of the article is organized as follows. In Section 2, the proposed method is introduced. Experiments and the evaluation results are presented in Section 3, followed by the concluding remarks in Section 4.

## 2 Materials and methods

In this problem, we have three type of input networks—drug–drug similarity network, disease–disease similarity network and drug–disease association network.

We constructed a single drug–drug similarity by combining multiple drug–drug similarity networks using SN^2^F (Section 2.2.1). We assume that $R=\{r_{1},r_{2},\ldots ,r_{m}\}$ is a set of drugs, where *m* is the number of drugs. Each edge in this network is a weighted edge that connects the two drugs.

Arbitration is made similarly for the disease–disease network. We constructed a single disease–disease similarity by combining multiple disease–disease similarity networks using SN^2^F (Section 2.2.1). We assume that $D=\{d_{1},d_{2},\ldots ,d_{n}\}$ is a set of disease, where *n* is the number of diseases. Each edge in this network is a weighted edge that connects the two diseases.

The drug–disease association network is modeled as a bipartite graph G(R,D,E), where E(G)⊆R×D is the set of edges between *R* and *D*. The adjacency matrix of this network *M_ij_* is such that if there is a known association between *r_i_* and *d_j_*, the value is equal to one (*M_ij_* = 1) and otherwise zero (*M_ij_* = 0).

**Fig. 1.**
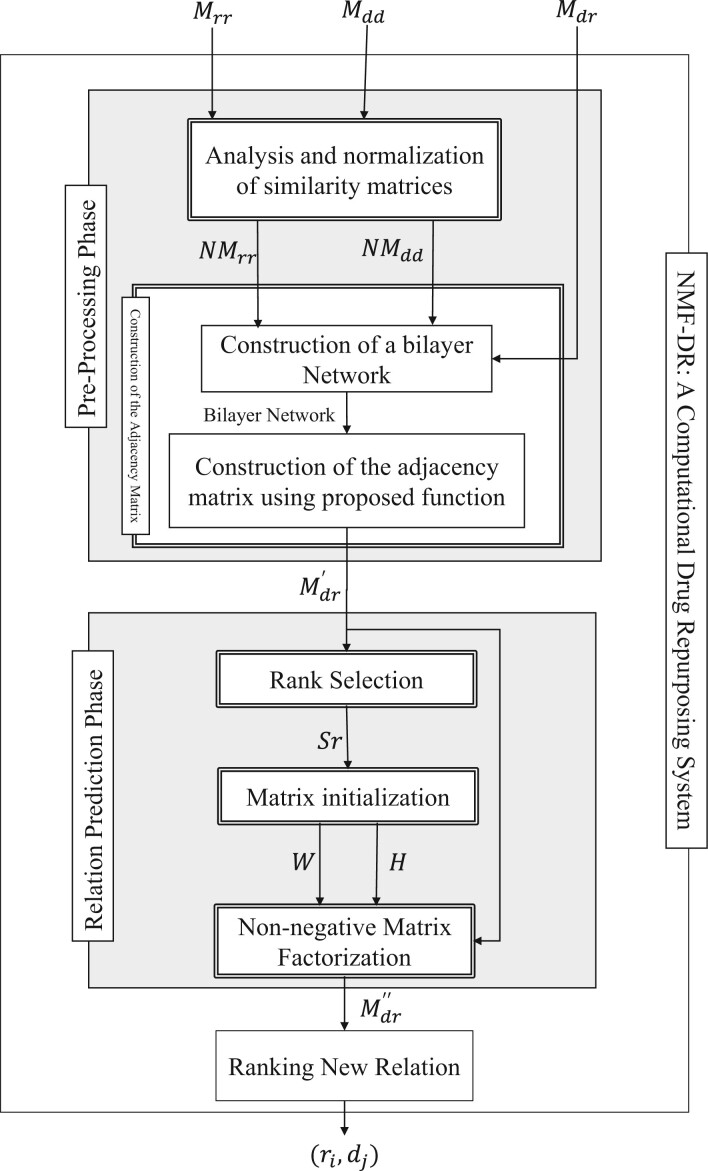
Block diagram of NMF-DR: a computational drug repurposing system

As shown in Figure  1, the drug–disease association matrix (*M_dr_*), drug similarity (*M_rr_*) and disease similarity (*M_dd_*), matrices are inputs of the NMF-DR method. New drug–disease associations between *i*th drug with *j*th disease $\left(r_{i},d_{j}\right)$ constitutes the output. 

Accordingly, NMF-DR involves two phases: the pre-processing phase and the relation prediction phase. In the first phase, an adjacency matrix is constructed, and in the second phase, new drug-disease relations are predicted. The description of abbreviations used in Figure  1 is presented in Table  1.

**Table 1.** Notations and variables used in this article

Symbol	Description
*M_dr_*	Drug–disease association matrix
$M_{rr}-M_{dd}$	Drug–drug and disease–disease similarity matrix
$NM_{dd}-NM_{rr}$	Normalized matrix
$M_{dr}^{\prime }$	Drug–disease adjacency matrix
Sr	Suitable rank
*W*, *H*	Low-rank matrices
$M_{dr}^{\prime \prime }$	Completed adjacency matrix
$SN^{2}F$	Similarity network normalization and fusion
WGC	Weighted graph construction
A-MDL	Accelerated minimum description length
A-HALS	Accelerated-hierarchical alternating least square
MU	Multiplicative updates
PG	Projected gradient

### 2.1 Pre-processing phase

#### 2.1.1 SN^2^F: analysis and normalization of similarity matrices

Research has shown that small similarities provide weak and poor information for predicting relationships (Luo *et al.*, 2016), which is why normalizing similarity matrices positively affect prediction systems’ ability. In this study, a normalization method is used to transform similarity matrices that are less informative for prediction into more informative similarity matrices.

We propose a method called SN^2^F that is derived from the Similarity Network Fusion (SNF) method (Wang *et al.*, 2014a). While SNF is used for fusing similarity matrices originally, we use the same algorithm for normalization purpose too. SNF can combine several types of similarity criteria that exist for a particular type of data. In this study, we use and modify the SNF algorithm not only for the purpose of fusing similarity matrices but also for normalization, and we called it SN^2^F. SNF resembles a network instance and then iteratively converts it to a single network based on the K-Nearest Neighbor (KNN) algorithm. These networks are combined in such a way that shared and type-dependent communications are available in the final network, and the final network most closely resembles all networks (Wang *et al.*, 2014a). The data fusion method used in this study is non-linear, which has the advantage of being able to have the same information along with the complementary information in different data types. It is also proposed to use this method as a normalizer of a similarity matrix to reinforce the large similarities and to zero the small similarities with a clustering-based approach derived from the KNN algorithm. The magnitude of each similarity value is calculated based on the initial similarity values of the edges throughout the network.

#### 2.1.2 WGN: construction of the heterogeneous network

Network-based CDR methods, in addition to nodes and edges, some methods utilize additional information such as the features of shared drug–target interactions. Using side information for link predictions, especially in sparse networks, can be very useful. Since graph structure information and side information represent various types of information as input to the predictor system, this information is expected to increase system performance (Menon and Elkan, 2011). Previous studies show that combining homogeneous information from multiple sources can lead to improved prediction accuracy.

In this study, drug similarity information, disease similarity and drug–disease relationship information are combined to form a heterogeneous network and then predict new drug–disease relationships by completing the neighborhood matrix of these heterogeneous data. Drug–drug similarity and disease–disease similarity networks by the drug–disease association network are combined to form a heterogeneous network.

This idea is derived from the basic structure of weighted MF and RW methods (Supplementary Fig. S2). According to this method we can say:

(1) $$M_{dr}{}^{\circ}(M_{dd}\cdot M_{dr}\cdot M_{rr})=M_{dr}^{\prime }$$

The equation uses matrix multiplication operation and Hadamard product (elementwise multiplication) to create $M_{dr}^{\prime }$. The weighted MF method is very popular for predicting missing entities. Since the subject under consideration is also a missing relationship prediction system (Wang and Zhang, 2013), it seems appropriate to use this idea as a method to combine similarity matrices. Although in this work instead of using the Kronecker product that was used in the original article we used matrix multiplication and pairwise multiplication to avoid creating a bigger matrix which has a bigger computational complexity than basic matrix product operation.

### 2.2 Relation prediction phase

We formulate the problem of drug repurposing as a drug–disease link prediction task. Link prediction problem is related to the missing value estimation problem solved in recommender systems (Zhang *et al.*, 2017) as well as in collaborative filtering methods (Sadeghi and Keyvanpour, 2019a). We use non-negative matrix factorization (NMF) method as mean of predicting missing drug disease association values in a bi-layered drug–disease association network. As we mentioned earlier, although, the use of RW-based methods are popular among methods used to predict the relationships between biological networks, especially in the case of DR (Luo *et al.*, 2018b, Wang *et al.*, 2014b, Luo *et al.*, 2016). MF-based methods have higher accuracy in comparison to RW-based methods, which makes this approach useful in this study (Luo *et al.* (2018a)). Also, MF-based methods are able to handle sparsity and scalability challenges (Bokde *et al.*, 2015). Therefore, methods based on the MF seem to be able to theoretically satisfy the purpose of this study.

In addition to the advantages of using matrix factorization algorithms, there are two important challenges to using matrix decomposition algorithms: (i) Choosing the right rank, and (ii) Appropriate initialization of decomposition matrices (Qiao, 2015). Therefore, in this research, an attempt is made to provide a system that can use appropriate ways to deal with these two basic challenges of these algorithms. This system is divided into three main sections: appropriate rank selection, initialization and matrix completion by matrix decomposition algorithms.

The observed drug–disease associations can be formulated as a bipartite network, and represented by a binary matrix $M_{dr}\in R^{n\times m}$, where *n* is the number of drugs, and *m* is the number of diseases. $M_{dr}{}_{ij}$ is the (*i*, *j*)th entry of *M_dr_*. If the drug vertex *r_i_* and the disease vertex *d_j_* are connected, $M_{dr}{}_{ij}=1$; otherwise $M_{dr}{}_{ij}=0$. NMF-DR factorizes the drug–disease association matrix *M_dr_* into two low-rank feature matrices $W\in R^{n\times k}$ and $H\in R^{m\times k}$, where *k* is the dimension of drug feature and disease feature in the low-rank spaces (Zhang *et al.*, 2018).

#### 2.2.1 A-MDL: rank selection in non-negative matrix factorization

NMF is a problem of estimating the non-negative matrix *X* as the product of two non-negative matrices. Suppose $X\in R_{>0}^{m\times n}$ and *m*, *n*, *r* are integers, then two matrices $W\in R_{>0}^{m\times r}$ and $H\in R_{>0}^{r\times n}$ are found such that X≈WH. The objective function is to minimize the Frobenius Norm of *X—WH*, that is:

(2) $$\begin{array}{c} \min||X-WH|{|}_{F}{}^{2} \end{array}$$

One of the primary parameters of NMF algorithms is the value of the matrix analysis rank. All known algorithms in this field depend on the predetermined value of the rank (the sizes of the new sub-matrices). In NMF, the sizes of sub-matrices have real meanings; in fact, this rank selects the number of extracted features. If the selected rank is too small, we may lose useful features, and if the rank is too large, we may model the noise. That is why choosing the right rank for both noise reduction, and modeling demonstrates the fundamentals of efficiency. In this study, we use a minimum descriptive length-based (MDL) method (Squires *et al.*, 2017) to find appropriate ranks. MDL is a method for selecting models with different complexities. The main idea of this MDL method is that the best model is a model that can compress the data best while preserving useful information as much as possible. Because the best way to compress data is to have the lowest cost of transmitting the message when the message is encrypted, the issue of the shortest message can be replaced with the lowest cost of message transmission.

In the NMF problem, the message is matrix *X*, which is estimated by the product of two matrices *W* and *H*. The model is simpler when rank is small, and consequently, *W* and *H* have few elements and therefore it is cheaper to encode. Yet, X≈WH will most likely be a bad approximation and will need additional information to improve this poor estimate (Squires *et al.* (2017)). The MDL principle is to choose the model that minimizes the total message length without any interest in how to optimally encode the message. By trading off the complexity and accuracy of the model, we hope to find the level of complexity that minimizes the transmission of noise while maximizing the transmission of real features. A-MDL is an accelerated version of Squires *et al.* (2017)) which address the high computational cost of this method by defining a threshold around the best rank computed in Luo *et al.* (2018a). DRRS (Luo *et al.*, 2018a) use a cross-validation approach which find the best rank with maximizing the Area Under Curve (AUC). This approach might lead to overfitting. Yet, NMF-DR address this challenge with using A-MDL method. This minor change in MDL method will not affect the final results since it does not change the algorithm significantly, yet it helps us to reduce the computation time.

#### 2.2.2 Multi-SVD: SVD-based initialization strategy for non-negative matrix factorization

The initialization of *W* and *H* matrices is crucial in the implementation of MF. Good initialization can induce faster convergence and better result of the optimization algorithm (Atif *et al.*, 2019).

Initialization can be carried out randomly, or can be improved by SVD. The initial estimate in this study is based on Singular value decomposition methods (SVD). The two well-known NNSVD and SVD-NMF methods, as well as the new NNSVD-LRC method, are applied alongside the randomized initialization method for this study, and then at each iteration, the best result (the method with the lowest error rate) is used as the initial value of the matrix decomposition algorithm (Supplementary Table S1). In most cases, the new NNSVD-LRC method will be able to achieve better results than other methods with relatively equal computational costs. Due to the complexity of NMF in this study, it is recommended to use several primary quantification methods and select the best result (Atif *et al.*, 2019).

#### 2.2.3 A-HALS: relation prediction using non-negative matrix factorization

As mentioned earlier, one of the challenges of using matrix analysis algorithms instead of RW models is the high computational cost of these algorithms. One way to deal with this challenge is to choose the right algorithm or improve available algorithms to increase the computational speed while maintaining their accuracy.

One of the safest of these algorithms is a method called alternating squares (ALS). The most important feature of the ALS method is mapping negative values to zero in each repetition of the algorithm(Berry *et al.*, 2007). This algorithm works by keeping *W* or *H* constant, the problem becomes the least squares with an infinite constraint problem (Ho, 2008). HALS is an improvement of ALS, which works by keeping the *H* constant while it successively updates each column of *W* with an optimal and easy-to-compute closed-form solution (Cichocki and Phan, 2009). Therefore, in this study, we use an improved speed method called A-HALS (Accelerated-Hierarchical Alternating Least Square) proposed by (Gillis and Glineur, 2012) as a matrix analysis optimization algorithm. The main idea of this method is to use the heaviest part of the HALS algorithm in such a way that the lighter parts of the algorithm are repeated only a limited number of (but reliable) times in each iteration (Gillis and Glineur, 2012). The A-HALS method is used in this study as a relationship prediction algorithm. According to this algorithm, by setting the stop conditions for the HALS algorithm, the convergence speed and prediction of these methods are increased.

## 3 Result

### 3.1 Datasets

In this article, we have used four gold standard datasets used in the CDR studies shown in Table  2 (Gottlieb *et al.*, 2011; Luo *et al.*, 2016; Martinez *et al.*, 2015; Wang *et al.*, 2013). We also show the results of each improvement that we have made in this study on the gold standard dataset (PREDICT dataset) obtained from (Gottlieb *et al.*, 2011). To be specific, drugs for all the mentioned datasets are collected from the DrugBank database (Wishart *et al.*, 2008). Diseases are collected from human phenotypes defined in the Online Mendelian Inheritance in Man (OMIM) database (Hamosh *et al.*, 2005). Although, DrugNet uses diseases annotated by Disease Ontology (DO) terms (Schriml *et al.*, 2019).

**Table 2.** The gold standard datasets used in this study

Datasets	Drugs (Registered By)	Diseases (Listed By)	Known Relations	Sparsity
PREDICT dataset (Gottlieb *et al.*, 2011)	593 (DrugBank database)	313 (OMIM database)	1933	$1.041^{-2}$
TL-HBGI dataset (Wu *et al.*, 2013)	1409 (DrugBank database)	5080 (OMIM database)	1461	$2.041^{-4}$
DrugNet dataset (Martinez *et al.*, 2015)	1490 (DrugBank database)	4516 (Disease Ontology) (Schriml *et al.*, 2019)	1008	$1.498^{-4}$
CDataset (Luo *et al.*, 2018a)	663 (DrugBank database)	409 (OMIM database)	2532	$9.337^{-3}$

Drug–drug similarity measures for PREDICT dataset (Gottlieb *et al.*, 2011) include: (i) drug chemical similarity; (ii) drug side effect similarity; (iii) sequence similarity; (iv) closeness in a PPI network; and (v) GO similarity measures. While, disease–disease similarity measures include: (i) phenotype similarity; and (ii) semantic phenotypic similarity measures.

For TL-HBGI dataset (Wu *et al.*, 2013), drug–drug similarities were calculated based on their chemical structures, a phenotype-based disease–disease similarity dataset downloaded from MimMine. For DrugNet dataset (Martinez *et al.*, 2015), Drug similarity is measured based on anatomical therapeutic chemical (ATC) codes, and disease similarity is measured based on Disease Ontology (DO) terms. Cdatasets is produced by combining DrugNet (Martinez *et al.*, 2015), and the gold standard dataset used in Luo *et al.* (2018a). In Cdatasets, drug similarity is measured based on chemical structures, and disease similarity is measured based on phenotypes using MimMiner. For more information on used datasets in this study see Supplementary Table S2.

### 3.2 Comparison and analysis of the results of the proposed system with other methods

We applied our NMF-DR method on four well-known benchmark datasets listed in Table  2, and reported cross-validated results in Table  3. Ten-fold cross-validation was used for the PREDICT, DrugNet and CDataSet datasets, whereas as we used 5-fold cross-validation for the TL-HGBI dataset (the original research that used TL-HBGI evaluated their model using 5-fold cross-validation only). Table  3 compares the AUC performance of NMF-DR with that of few existing repurposing methods applied on the same datasets. Additional comparison results also appear in the Supplementary Table S1. NMF-DR significantly outperforms all the other methods across all datasets, reaching AUC results of 0.97 on PREDICT data, 0.98 on both DrugNet and CDataSet data and 0.99 on TL-HBGI data.

**Table 3.** The Performance of different methods on different datasets

	**AUC**			**AUC**
	10-fold cross validation			5-fold cross validation
Method	PREDICT Dataset	DrugNet Dataset	CDataSet	TL-HGBI Dataset
HGBI (Wang *et al.*, 2013)	0.82	—	0.85	—
TL-HGBI (Wang *et al.*, 2014b)	—	—	—	0.95
DrugNet (Martinez *et al.*, 2015)	0.77	0.94	0.8	—
MBiRW (Luo *et al.*, 2016)	0.91	0.95	0.93	—
RWHNDR(Luo *et al.*, 2018b)	0.92	—	0.94	—
NTSIM (Zhang *et al.*, 2017)	—	—	—	0.96
DRRS (Luo *et al.*, 2018a)	0.93	0.93	0.94	—
ANMF (Yang *et al.*, 2019)	0.93	—	0.95	—
KBMF (Gönen *et al.*, 2013)	0.91	—	0.92	—
MSBMF (Yang *et al.*, 2021)	0.94	—	0.95	—
SCMFDD (Zhang *et al.*, 2018)	—	—	—	0.97
PREDICT (Gottlieb *et al.*, 2011)	0.89	—	—	—
PreDR (Wang *et al.*, 2013)	0.86	—	—	—
SMKF (Moghadam *et al.*, 2016)	0.91	—	—	—
**NMF-DR**	**0.97**	**0.98**	**0.98**	**0.99**

The best methods and results are indicated in bold.

Since different similarity matrices were available for PREDICT dataset, we could test our method with all the steps in NMF-DR method including fusion and normalization of all similarity matrices using SN^2^F. In this comparison, the power of MF methods such as NMF-DR and DRRS can also be demonstrated against RW methods such as RWHNDR and MBiRW as well as learning-based methods such as PREDICT.

One of the important points in this article is that, despite the varying degree of sparsity of the mentioned datasets, NMF-DR was able to perform reasonably well and does not reduce the ability of the method. It can also be said that the NMF-DR is also able to maintain its accuracy with increased size of the networks built in the pre-processing section.

NMF-DR also outperforms learning-based methods, including PreDR (Wang *et al.*, 2013), PREDICT (Gottlieb *et al.*, 2011) and SKMF (Moghadam *et al.*, 2016). Due to the class imbalance problem, here F-measure are used instead of AUC. As mentioned earlier, the existing datasets in drug repurposing suffers from class imbalances challenge, so criteria such as the F-measure and AUPR are more suitable and informative than other evaluation criteria, such as accuracy and AUC. NMF-DR shows higher AUC and Accuracy, for learning-based methods, we also used F-measure to evaluate our method. Since, most repurposing studies only report AUC performances and not AUPR performances on given datasets, we have decided to only report AUC results in this research.

### 3.3 Analyzing the impact of our proposed improvements in the NMF-DR

To evaluate the performance of NMF-DR and analyze the effect of our proposed improvements in each step of the NMF-DR system we conducted the following experiments.

Different criteria can affect the performance of a data integration method. One of the most effective metrics in the performance of an integration method is the measure of variability between data. According to this assertion and the results obtained, the efficiency of using different biological side information is shown in Table  4. Accordingly, the performance of the method is improved as different biological information is used to predict and integrate it (Luo *et al.*, 2018a). This section examines the diversity of resources used in this study, namely, similarity matrices made from the characteristics of drugs and diseases. Based on the evaluation in Table  4, it is shown that the AUC value of the pre-processing step with the SN^2^F normalization increases. Accordingly, combining drug similarity based on side effects and disease similarity based on phenotypes semantic similarity has been the best set of side information to predict drug–disease relationships, which means that this set of similarities have more meaningful information than any other set of similarities. Table  4b also shows SN^2^F normalization method impact the results of NMF-DR. Based on the results obtained, it can be seen that this method shows better results on those side information similarity matrices that contain less useful information than those with more useful information.

**Table 4.** Pre-processing phase: a comparative view of the composition of different similarity measures with and without SN^2^F in the proposed NMF-DR framework on PREDICT dataset (AUC)

**a. Without SN^2^F**		
Drug similarities	Disease Similarities	
	Phenotype similarity	Semantic phenotypic similarity
Drug chemical similarity	0.909018	0.914015
Drug side effect similarity	**0.91377**	**0.917137**
Closeness in a PPI network	0.905549	0.911368
GO similarity	0.908694	0.91467
Sequence similarity	0.879688	0.890363
	**b. With SN^2^F**	
Drug similarities	Disease Similarities	
	Phenotype similarity	Semantic phenotypic similarity
Drug chemical similarity	0.914314	0.916921
Drug side effect similarity	0.91585	0.917676
Closeness in a PPI network	0.915723	0.912903
GO similarity	0.914404	0.91481
Sequence similarity	**0.916013**	**0.918274**
**Fusion similarities**	**0.918369914**	

The best methods and results are indicated in bold.

For example, the combination of sequence-based drug similarity data and phenotype-based disease similarity data, which had the lowest AUC in Table  4a, would make the most changes after normalization, and this growth would mean that our goal to normalize and eliminate weak information and to empower stronger information to improve method performance works perfectly.

In this section, in addition to the results reported in Table  4, the results of combining existing similarity criteria for the PREDICT dataset with the single-use model of a similarity criterion are calculated. In theory, the more varied the information is, the better the performance of the system. The observations here confirm this, but the important point in this section is whether the difference between the two models worth dealing with the computational cost of the feature combination?

To answer this question, it can be said that although the difference between these two values is not significant, one should note two points: (i) the reported value without SN^2^F algorithm represents the better reports. Therefore, the selection of the best similarity criteria for the proposed system is very important because, as shown in Table  4, lower values such as 0.87 are also in this comparison, indicating the importance of selecting the appropriate similarity criterion. (ii) The reported value for combining similarity measures depends entirely on the number of similarity measures used as well as the input similarity measures itself. Therefore, although the effect of this method on combining values does not appear to be high, one can understand the value of using this method based on the results in Table  4.

As for the prediction phase, the selection of a suitable rank for MF has paramount importance, and many methods have been proposed to discover the merits and disadvantages of each method. In this study, a method based on the minimum description length is used, since this method will be very time consuming, we proposed an accelerated form of MDL model called A-MDL, which calculates only a fraction of the possible values for the MF rank. This proposed method will not affect accuracy but can be very effective in the reducing computational time of implementation.

As shown in Table  5a, selecting the appropriate rank will have a significant impact on the outcome of the proposed system. In fact, it can be said that facing the challenge of finding the best rank will have the most impact on our system, and this will be the most important part of the proposed system (Santiso *et al.*, 2019). As mentioned earlier, a small rank value can reduce the accuracy of the method because it may lose useful information in this matrix analysis while larger rank, along with raising the computational cost may cause overfitting.

**Table 5.** Prediction phase: a comparative overview of the results of each step of NMF-DR method on PREDICT dataset

**a. The impact of our proposed rank selection step: A-MDL**	
Method	AUC
Default rank	0.9153593
Pre-Processing phase + default rank	0.91837
A-MDL selected rank	0.969322
Pre-Processing phase + A-MDL selected rank	**0.9700965**
**b. The impact of our proposed initialization step: Multi-SVD**	
Method	AUC
Random initialization	0.9153593
Multi-SVD initialization	0.919802
Default rank+ multi-SVD	**0.9721509**
Pre-Processing+ default rank +random initialization	0.914306
Pre-Processing + default rank + multi-SVD initialization	0.917966
Pre-Processing +selected r using A-MDL+ random initialization	0.9701681
Pre-Processing + selected r using A-MDL + multi-SVD initialization	0.9715765
**c. The impact of using different matrix factorization method**	
Methods	AUC
ALS	0.9153593
HALS	0.9715765
A-HALS	**0.972179**

The best methods and results are indicated in bold.

**Fig. 2.**
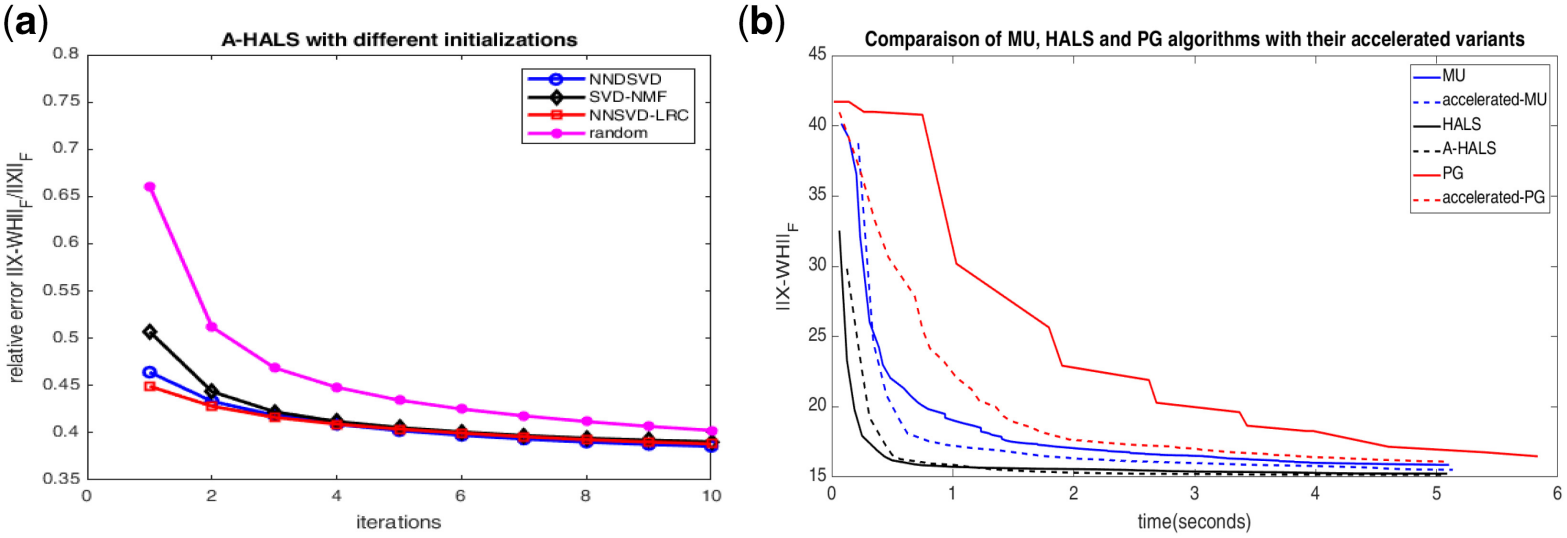
(**a**) A demonstration of the impact of SVD-based initialization methods compared with other methods on PREDICT dataset. (**b**) Comparisons of matrix decomposition algorithms and their accelerated versions on PREDICT dataset

Next, we tested our proposed initialization method. Since the main idea of the proposed method of this research is to choose the best value out of the four well-known methods (NNDSVD, SVD-NMF, NNSVD-LRC and Random initialization), in each implementation, one should choose the one that has the least relative error rate, as shown in Figure  2a. The relative error values of each method are very close to each other. It is expected that the accuracy values of each method will be very close to each other. For this purpose, we used 10 for iteration because after 10 repetitions, the results converge, and there is no significant change afterward.

As expected, if the initialization methods and the proposed initialization methods of this study were separately applied to the selected dataset, the accuracy of these methods would be as follows in Table  6. Table  6 and Figure  2a provide a demonstration of how the initialization of the decomposition matrices affects the proposed NMF-DR framework. The impact of Multi-SVD method for initialization in our proposed framework can be seen in Table  5b. The figures in Table  5b show that using the proposed Multi-SVD method can improve the result of the framework in comparison to random matrix initialization. We also examined whether using a random rank also can affect the initialization method.

**Table 6.** The comparison of the proposed initialization method (Multi-SVD) with some of the existing initialization methods based on AUC (PREDICT dataset)

Initialization methods	AUC
NNDSVD	0.9689702
SVD-NMF	0.9685116
NNSVD-LRC	0.9672019
Random	0.9671383
Multi-SVD	**0.9691728**

The best methods and results are indicated in bold.

And finally for our final step of prediction, we first do a comparison of some well-known methods in the field of matrix decomposition. Figure  2b compares the performances of three decomposition methods, MU (Lee and Seung, 1999), PG (Lin, 2007) and HALS (Ho, 2008) along with the accelerated versions (Gillis and Glineur, 2012). This comparison shows that the accelerated version of the methods not only increased the speed but also showed better convergence. It can also be seen that the HALS method has the fastest convergence compared to the other two methods, and the A-HALS has the least final error rate (Table  7).

**Table 7.** Comparison of MU, HALS and PG algorithms and their accelerated model for final error on PREDICT dataset

Method	Final error
MU	15.867820
Accelerated MU	15.520418
HALS	15.242294
Accelerated HALS	**15.159298**
PG	16.475821
Accelerated PG	16.069888

The best methods and results are indicated in bold.

In addition to Figure  2b, the final error result of each method can be seen in Table  7. In the following, the results of applying the A-HALS method to PREDICT data are discussed. According to Table  5c, two other models are significantly improved compared with the basic form that is implemented (only using the ALS integration and implementation stage for prediction). Also, as can be seen by applying the accelerated modeling of the HALS algorithm, the system was able to maintain its performance reliably and not only did not decrease performance but also increased the accuracy slightly.

### 3.4 Comparison of NMF-DR with other matrix factorization-based methods

Since choosing a suitable rank of the factorization and providing a good initialization method is a challenging task in MF-based models, previous MF-based studies (Luo *et al.*, 2018a, Zhang *et al.*, 2018; Yang *et al.*, 2021) tried to address at least one of these challenges. In this study, we address these two challenges while maintaining the accuracy. Comparing MF-based methods (DRRS (Luo *et al.*, 2018a), ANMFF (Yang *et al.*, 2019), KBMF (Gönen *et al.*, 2013), MSBMF (Yang *et al.*, 2021) and SCMFDD (Zhang *et al.*, 2018)) with NMF-DR at Table 3 shows that NMF-DR obtains AUC value of 0.97 and 0.98, respectively, and outperforming all other methods significantly.

### 3.5 Evaluation of NMF-DR with proposed practical criteria

The proposed system is related to the network-based approach, and the subcategory of MF. These approaches that are used in this study are capable of dealing with heterogeneous data, and they do not fail to address the sparsity as well as scalability. The set of experiments in Section 3.2 confirms this claim. On the other hand, other methods sometimes have difficulty in facing such challenges as sparsity and do not provide acceptable results. However, high computational complexity, failure to specify multiple parameters make it difficult to use MF-based methods in more complex networks.

### 3.6 Case study: computationally identified approved drugs for breast cancer

In addition to the cross-validation experiments, we also applied NMF-DR on all the collected data to make novel drug usage predictions. In this article, we present the results of our method for breast cancer disease, and our top 5 ranked predictions are as follows: Leuprolide (DB00007), Estramustine (DB01196), Flutamide (DB00499), Bicalutamide (DB01128) and Mitoxantrone (DB01204).

Breast cancer is one of the most important causes of mortality in women. According to the World Health Organization, breast cancer is the most common cancer among women, affecting more than 5.1 million people each year. Also, the highest number of cancer deaths among women is related to breast cancer (Salehiniya *et al.*, 2018; Siegel *et al.*, 2019). One of the most effective ways to reduce mortality and reduce costs is to predict early disease as well as initiate timely treatment. DR can also be very effective in the treatment sector by providing appropriate drugs at the fastest time and at the lowest cost for patients who need medicines (Aggarwal *et al.*, 2021). The prediction results are confirmed based on some public databases, current clinical trials and literature (Supplementary Table S3). We find that some top-ranked predictions have been confirmed by existing researches. It is reported that drugs known to treat prostate cancer can also be used for breast cancer. Further research shows that although breast and prostate cancer occur in two different regions of the body of men and women, they are both biologically and genetically similar because they are both hormone dependent (Risbridger *et al.*, 2010). These successful prediction instances further confirm that NMF-DR has the potential to predict novel drugs for disease indications.

## 4 Discussions and conclusions

In the CDR field, although several useful studies have been introduced, there are still some challenges in this area. Hence, in this article, to improve existing CDR methods, a novel network-based framework called NMF-DR proposed and compared with other methods. Theoretically and practically, we show that NMF-DR is superior to the some well-known existing drug repurposing methods as we adopt a MF-based method on a network to capture complex topological patterns across different data sources.

In summary, our findings suggest that network-based recommender systems can be beneficial to explore the relationships of heterogeneous drug–disease networks for CDR purposes. The network-based model developed here can help discover novel and effective treatments for multiple complex diseases if broadly implemented. We also noticed that there is much room to improve our method. In the following, there are other possible ways to improve the proposed methods.

For example, it seems that besides the use of drugs and diseases information, the target information and other relevant biomedical information can improve the detection of new drug-disease relationships by devising new network-based methods which can integrate multiple types and multiple sources of omics data appropriately. Also, one of the most important ideas for the future development of the proposed system is to improve the feature extraction and prediction process. Heterogeneous network embedding methods can be used for feature extraction and link prediction purpose between drugs and diseases. In addition, the use of the MF method applied in this study has limitations and challenges to be addressed in the future. These challenges can be attributed to the high cost of computing the appropriate initial parameters. NMF-DR approach can also be used to predict other relationships between biological entities such as drug-protein or miRNA-drug associations, for instance. The fourth idea is to improve the run time of predicting the drug-disease associations in the proposed system. Since the proposed systems approach is a time-consuming MF approach, reducing run time in this section can be very useful.

There are other methods for setting the parameters of NMF-based methods. For example, one can use the dispersion coefficient for rank selection. As one of our future works, we can propose new methods and also conduct an experiment for showing the importance of finding the best rank and setting other parameters. Finally, using these computational methods for new challenging diseases such as COVID-19, can be extremely helpful to handle crisis in shorter time.

## Funding

This work was supported by the National Science and Engineering Research Council of Canada (NSERC) Discovery Grants #RGPIN-2016-05017 (Alioune Ngom) and #RGPIN-2019-05350 (Jianguo Lu).

*Conflict of Interest*: none declared. 

## Supplementary Material

btab826_Supplementary_Data
