# Supplementary material for: A network-based drug repurposing method via non-negative matrix factorization
Source: Bioinformatics. 2021 Dec 7;38(5):1369–77. doi: 10.1093/bioinformatics/btab826 (PMC8825773; doi:10.1093/bioinformatics/btab826)
Supplement: btab826_Supplementary_Data [file btab826_supplementary_data.pdf]

A Network-Based Drug Repurposing Method Via Non-Negative Matrix Factorization (Supplementary File)

Shaghayegh Sadeghi, Jianguo Lu, Alioune Ngom

Reviewing the proposed methods in computational drug repurposing showed that computational drug repurposing methods can be divided into two main categories: network-based drug repurposing methods and learning-based drug repurposing (Supplementary Figure S1)

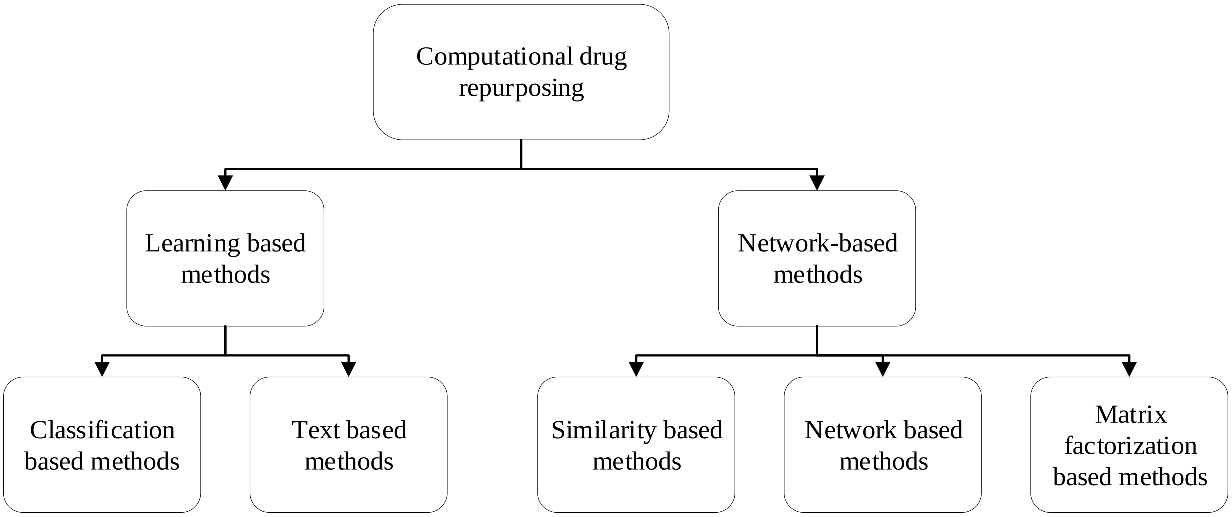

Fig. S1. Approaches classification for computational drug repurposing[1]

Drug-drug similarity network, disease-disease similarity network, and drug-disease association network are combined to form a heterogeneous network and then predict new drug-disease association by completing the neighborhood matrix of these heterogeneous data. The heterogeneous network is constructed by connecting the two drug-drug and disease-disease networks by the drug-disease association network.

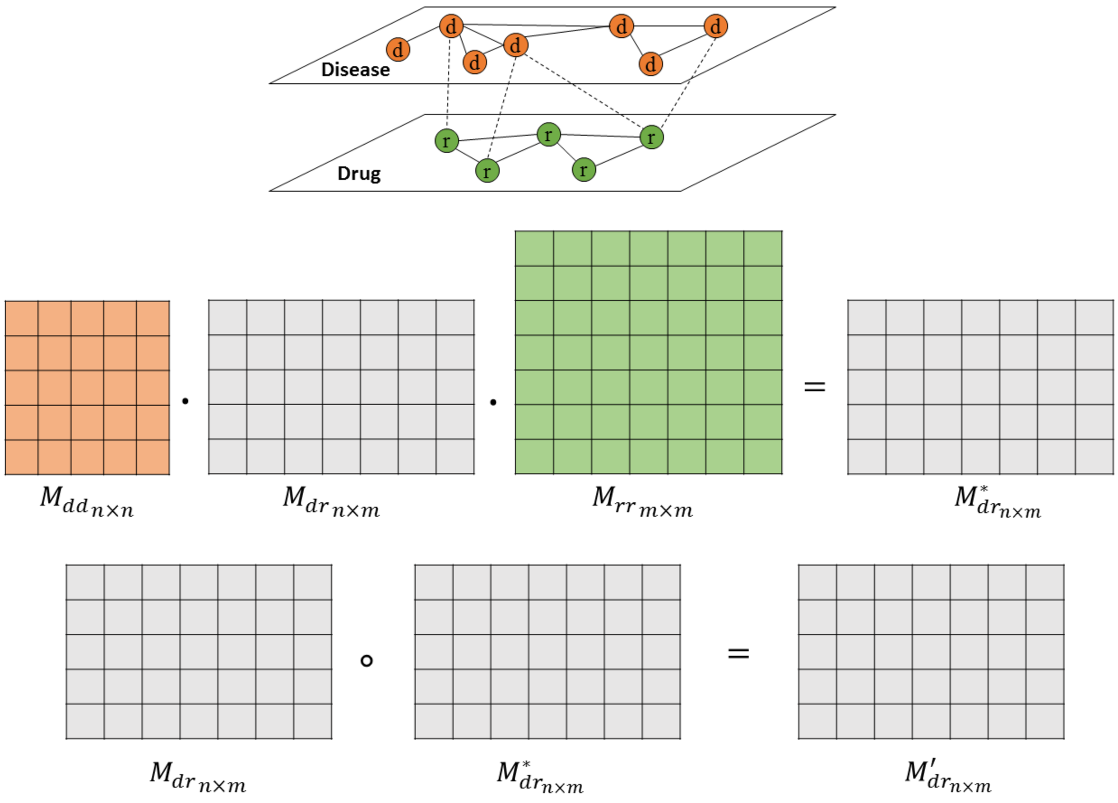

Fig. S2. The heterogeneous drug-disease network and its adjacency matrix [2]

The drug-disease network is modelled as a bipartite graph $G(R;\ D;\ E)$ where R is the set of drug vertices, D is the set of disease vertices, and where $E(G)\ \subseteq\ R \times D$ is the set of edges such that an edge $(r_i,\ d_j)\ \in\ E(G)$ has an initial weight value $e_{ij}\ =\ 1$ if there is a known association between r_i and d_j, and a weight value $e_{ij}\ =\ 1$ otherwise.

				...	
	0	1	0	...	0
	0	0	1	...	0
	1	0	1	...	1
...	...	...	...	...	...
	0	0	1	...	0

Fig. S3. Drug and Disease relation matrix, rows represent diseases and columns represent drugs

A study on initialization methods for matrix factorization is presented. Based on this study, random initialization methods can make NMF suffer from slow convergence, and hence the whole computational process can become very costly [3]. Also, random initalization may converge to a local minimum, so it becomes necessary to run several instances of the algorithm using different random initializations and then select the best solution [4].

Table S1 :A Study on Initialization Methods for Matrix factorization

Method	Benefits	Challenges
SVD-NMF [3]	●Relatively easy to implement<br>●Low computational complexity<br>●High convergence speed	○The error initialization does not decrease with increasing rank<br>○In some cases, the results of random methods are better than this
NNSVD [4]	●High convergence speed<br>●Low convergence error	○The error initialization does not decrease with increasing rank<br>○In some cases, the results of random methods are better than this<br>○Creates high-density decomposition matrices
NNSVD-LRC [5]	●Production of sparse matrices and memory retention<br>●More resistance to noise than previous methods<br>●As the rank increases, the error of initialization decreases	○The high complexity of implementation<br>○In some cases, the results of random methods are better than this
Random	●Easy to implement<br>● Higher result by chance	○ Does not have any resistance to noise<br>○The error initialization does not decrease with increasing rank<br>○Converge to a local minimum may happen<br>○Slow convergence

In this study, we have used four gold standard data sets used in the drug repurposing studies shown in Table S2 (Gottliebet al., 2011 , Luoet al.,2016 , Wanget al., 2013, Martinezet al., 2015).

Table S2. The Gold Standard Datasets Used In This Stud					
Data set	Drugs (Registered By)	Diseases (Listed By)	Known Relations	Sparsity	Description
PREDICT Dataset	593 (DrugBank database)	313 (OMIM database)	1933	1.041^{-2}	Drug-drug similarity measures used include: (1) Chemical similarity; (2) Side effect-based similarity; (3) Sequence-similarity; (4) Closeness in a PPI network; (5) GO-based.<br>Disease–disease similarity measures used include: (1) Phenotype similarity; (2) Semantic phenotypic similarity
TL-HGBI Dataset	1409 (DrugBank database)	5080 (OMIM database)	1461	2.041^{-4}	Drug-drug similarity measures used include Chemical similarity<br>Disease–disease similarity measures used include Phenotype similarity
DrugNet Dataset	1490 (DrugBank database)	4516 (Disease Ontology (DO))	1008	1.498^{-4}	Drug-drug similarity measures used include Chemical-based<br>Disease–disease similarity measures used include Phenotype similarity
CDataset	663 (DrugBank database)	409 (OMIM database)	2532	9.337^{-3}	Drug-drug similarity measures used include Chemical similarity<br>Disease–disease similarity measures used include Phenotype similarity

In addition to the cross-validation experiments, we also applied NMF-DR on all the collected data to make novel drug usage predictions. In this paper, we present the results of our method for Breast Cancer disease, and our top 5 ranked predictions can be found in Supplementary Table S3. Breast cancer is one of the most important causes of mortality in women. According to the World Health Organization, breast cancer is the most common cancer among women, affecting more than 5.1 million people each year. Also, the highest number of cancer deaths among women is related to breast cancer [6, 7]. One of the most effective ways to reduce mortality and reduce costs is to predict the early onset of the disease as well as initiate timely treatment. Drug repurposing can also be very effective in the treatment sector by providing appropriate drugs at the fastest time and at the lowest cost for patients who need medicines [8]. The prediction results are confirmed based on some public databases, current clinical trials, and literature. We find that some top-ranked predictions have been confirmed by existing researches. It is reported that drugs known to treat prostate cancer can also be used for breast cancer. Further research shows that although breast and prostate cancer occur in two different regions of the body of men and women, they are both biologically and genetically similar because they are both hormone dependent [9]. These successful prediction instances further confirm that NMF-DR has the potential to predict novel drugs for disease indications.

Table S3:Case study results: the top 5 predictions for the selected disease

Drug ID	Drug Name	Proof	Initial Indication	Chemical Structure
DB00007	Leuprolide	DrugBank	For the treatment of prostate cancer, endometriosis, uterine fibroids, and premature puberty	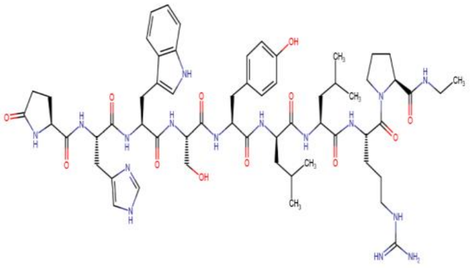
DB01196	Estramustine	clinicaltrials.gov	For the palliative treatment of patients with metastatic and/or progressive carcinoma of the prostate	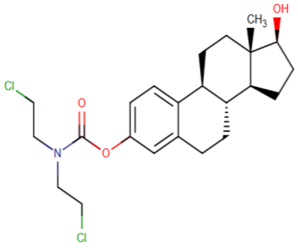
DB00499	Flutamide	A clinical study [10]	For the management of locally confined Stage B2-C and Stage D2 metastatic carcinoma of the prostate	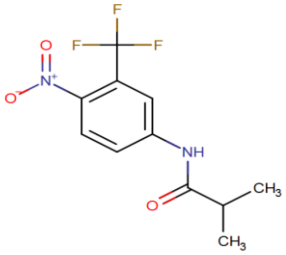

DB01128	Bicalutamide	clinicaltrials.gov	For treatment (together with surgery or LHRH analogue) of advanced prostatic cancer.	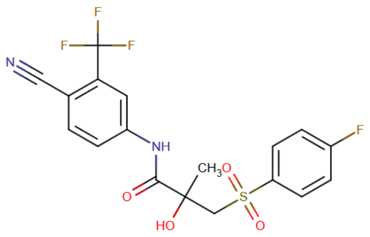
DB01204	Mitoxantrone	Drugbank	For the treatment of secondary (chronic) progressive, progressive relapsing, or worsening relapsing-remitting multiple sclerosis	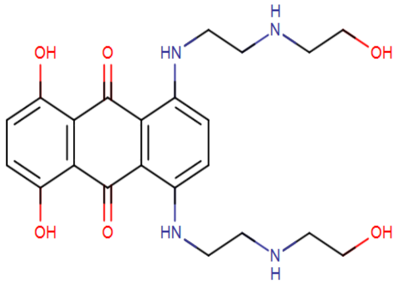

References

[1] Sadeghi, S. S. and Keyvanpour, M. R. (2020). Computational drug repurposing: Classification of the research opportunities and challenges. *Current Computer Aided Drug Design*, **16**(4), pages 354–364.

[2] Sadeghi, S. S. and Keyvanpour, M. (2019a). RCDR: a recommender based method for computational drug repurposing. In *2019 5th Conference on Knowledge Based Engineering and Innovation (KBEI)*, pages 467–471. IEEE.

[3] H. Qiao,(2015). "New SVD based initialization strategy for non-negative matrix factorization," *Pattern Recognit. Lett.*, vol. 63, pages 71–77.

[4] C. Boutsidis and E. Gallopoulos,(2008) "SVD based initialization: A head start for non-negativematrix factorization," *Pattern Recognit.*, vol. 41, no. 4, pages 1350–1362.

[5] S. M. Atif, S. Qazi, and N. Gillis,(2019). "Improved SVD-based initialization for non-negative matrix factorization using low-rank correction," *Pattern Recognit. Lett.*, vol. 122, pages 53–59.

[6] Salehiniya, H., Haghighat, S., Parsaeian, M., Majdzadeh, R., Mansournia, M., and Nedjat, S. (2018). Iranian breast cancer risk assessment study (irbcras): a case control study protocol. WCRJ, 5, 1–5.

[7] Siegel, R. L., Miller, K. D., and Jemal, A. (2019). Cancer statistics, 2019. CA: a cancer journal for clinicians, 69(1), 7–34.

[8] Aggarwal, S., Verma, S. S., Aggarwal, S., and Gupta, S. C. (2021). Drug repurposing for breast cancer therapy: Oldweaponfor new battle. In Seminars in cancer biology, volume 68, pages 8–20. Elsevier.

[9] Risbridger, G. P., Davis, I. D., Birrell, S. N., and Tilley, W. D. (2010). Breast and prostate cancer: more similar than different. Nature Reviews Cancer, 10(3), 205–212.

[10] D. J. Perrault, D. M. Logan, D. J. Stewart, V. H. C. Bramwell, A. H. G. Paterson, and E. A. Eisenhauer,(1988). Phase II study of flutamide in patients with metastatic breast cancer. A National Cancer Institute of Canada Clinical Trials Group study, *Invest. New Drugs*, **6**(3), pages 207–210.
